# Physiological Responses to Two Hypoxic Conditioning Strategies in Healthy Subjects

**DOI:** 10.3389/fphys.2016.00675

**Published:** 2017-01-10

**Authors:** Samarmar Chacaroun, Anna Borowik, Shawnda A. Morrison, Sébastien Baillieul, Patrice Flore, Stéphane Doutreleau, Samuel Verges

**Affiliations:** ^1^HP2 Laboratory, University Grenoble AlpesGrenoble, France; ^2^U1042, Institut National de la Santé et de la Recherche MédicaleGrenoble, France; ^3^Applied Kinesiology, University of PrimorskaPrimorska, Slovenia; ^4^Grenoble Alpes University HospitalGrenoble, France

**Keywords:** hypoxic conditioning, sustained hypoxia, intermittent hypoxia, blood pressure, heart rate variability, tissue oxygenation

## Abstract

**Objective:** Hypoxic exposure can be used as a therapeutic tool by inducing various cardiovascular, neuromuscular, and metabolic adaptations. Hypoxic conditioning strategies have been evaluated in patients with chronic diseases using either sustained (SH) or intermittent (IH) hypoxic sessions. Whether hypoxic conditioning via SH or IH may induce different physiological responses remains to be elucidated.

**Methods:** Fourteen healthy active subjects (7 females, age 25 ± 8 years, body mass index 21.5 ± 2.5 kg·m^−2^) performed two interventions in a single blind, randomized cross-over design, starting with either 3 x SH (48 h apart), or 3 x IH (48 h apart), separated by a 2 week washout period. SH sessions consisted of breathing a gas mixture with reduced inspiratory oxygen fraction (FiO_2_), continuously adjusted to reach arterial oxygen saturations (SpO_2_) of 70–80% for 1 h. IH sessions consisted of 5 min with reduced FiO_2_ (SpO_2_ = 70–80%), followed by 3-min normoxia, repeated seven times. During the first (S1) and third (S3) sessions of each hypoxic intervention, cardiorespiratory parameters, and muscle and pre-frontal cortex oxygenation (near infrared spectroscopy) were assessed continuously.

**Results**: Minute ventilation increased significantly during IH sessions (+2 ± 2 L·min^−1^) while heart rate increased during both SH (+11 ± 4 bpm) and IH (+13 ± 5 bpm) sessions. Arterial blood pressure increased during all hypoxic sessions, although baseline normoxic systolic blood pressure was reduced from S1 to S3 in IH only (−8 ± 11 mmHg). Muscle oxygenation decreased significantly during S3 but not S1, for both hypoxic interventions (S3: SH −6 ± 5%, IH −3 ± 4%); pre-frontal oxygenation decreased in S1 and S3, and to a greater extent in SH vs. IH (−13 ± 3% vs. −6 ± 6%). Heart rate variability indices indicated a significantly larger increase in sympathetic activity in SH vs. IH (lower SDNN, PNN50, and RMSSD values in SH). From S1 to S3, further reduction in heart rate variability was observed in SH (SDNN, PNN50, and RMSSD reduction) while heart rate variability increased in IH (SDNN and RMSSD increase).

**Conclusions:** These results showed significant differences in heart rate variability, blood pressure, and tissue oxygenation changes during short-term SH vs. IH conditioning interventions. Heart rate variability may provide useful information about the early adaptations induced by such intervention.

## Introduction

Although chronic hypoxia is known to be an aggravating factor in several cardiovascular and respiratory diseases, hypoxic exposure may also be of significant benefits to one's cardiorespiratory status as demonstrated in animal models (e.g., Béguin et al., 2005) and as reviewed recently by our group (Verges et al., 2015) and others (Almendros et al., 2014; Navarrete-Opazo and Mitchell, 2014; Mateika et al., 2015). Hypoxic exposure as a potential therapeutic aid is a promising rehabilitation method for many clinical populations, including hypertensive (Serebrovskaya et al., 2008) and overweight (Kayser and Verges, 2013) patients. Unlike in chronic respiratory failure where hypoxia is most often associated with hypoventilation and hypercapnia, the combination of hypoxia, hyperventilation (and slight resultant hypocapnia) can be used to exploit certain physiological benefits of hypoxic exposure e.g., decreased blood pressure observed in animal models and hypertensive patients after repeated hypoxic exposures (Serebrovskaya et al., 2008). Indeed, exposure to either normobaric or hypobaric hypoxia has historically been implemented to improve athletic performance through various physiological mechanisms, including increased hemoglobin mass and enhanced muscle capilarization and metabolic capacities (reviewed in Millet et al., 2010). From this perspective, and using methods previously employed in sport medicine, repeated hypoxic exposures, i.e., hypoxic conditioning, may be seen as a new preventive, and therapeutic strategy, especially for deconditioned individuals.

Unfortunately, current research in the field of hypoxic conditioning does not provide the optimal hypoxic exposure required to obtain protective or therapeutic effects. It is now recognized that the consequences of hypoxic exposure depend on a dose–response relationship ranging from normoxia to deleterious severe hypoxia [intermittent inspiratory oxygen fraction (FiO_2_) < 0.08–0.10 and arterial oxygen saturation (SpO_2_) <70%, with short cycles <2 min], with intermediate doses (moderate hypoxia, FiO_2_ > 0.10 or SpO_2_ ≥ 70–80%) and patterns (intermittent hypoxia with >5-min cycles or sustained hypoxia) of hypoxia that can have positive cardiovascular and metabolic effects (Navarrete-Opazo and Mitchell, 2014; Verges et al., 2015). Previous studies on hypoxic conditioning in healthy subjects and patients used two main types of hypoxic exposure, with repetitive sessions of either intermittent [IH, e.g., 3–7 min hypoxia and 3–5 min normoxia, repeated 3–10 times (Bernardi et al., 2001a; Burtscher et al., 2004; Lyamina et al., 2011; Zhang et al., 2014)] or sustained [SH, e.g., 1 h under sustained hypoxia (Rodríguez et al., 1999; Katayama et al., 2001, 2009; Lusina et al., 2006)] hypoxia. Whether these two types of hypoxic conditioning may induce different adaptations needs to be elucidated in order to establish optimal hypoxic conditioning strategies.

The possible deleterious side-effects of hypoxia [cardiovascular and metabolic dysfunctions as observed in sleep apnoea syndrome for instance: increased blood pressure, impaired glucose, and lipid metabolism (Lévy et al., 2011)] have to be considered when applying hypoxic conditioning strategy, especially to higher-risk, clinical populations. The hypoxic dose and pattern used for hypoxic conditioning should probably be adapted depending on patient characteristics and individual responses. The determination of the optimal hypoxic dose should occur during the beginning of the hypoxic conditioning program (i.e., over the first sessions) in order to avoid either insufficient (and therefore no treatment effect) or too severe (and its potential deleterious side-effects) hypoxic stimuli. Monitoring the efficiency and safety of hypoxic conditioning strategies requires to identify physiological biomarkers reflecting the early responses (during the first session and over the following sessions) to hypoxic conditioning.

Acute hypoxia induces within the first minutes and hours of exposure typical cardiorespiratory and autonomic nervous system responses including hyperventilation, tachycardia, and increased sympathetic activity. These mechanisms have been especially studied in the context of acute and chronic altitude exposure, i.e., prolonged sustained hypoxia (several hours and days staying at altitude, Mazzeo, 2008). While some of these mechanisms may be helpful in monitoring the effect of hypoxic conditioning strategies, few data are available in the literature regarding the main physiological responses during and after repetitive SH or IH conditioning sessions. Changes in blood pressure and autonomic nervous system are potential relevant markers of the physiological changes induced by repetitive hypoxic exposure. Severe intermittent hypoxia as encountered in sleep apnoea alters baroreflex activity and increases blood pressure (Lévy et al., 2011) while hypoxic conditioning strategy may improve baroreflex sensitivity (Haider et al., 2009) and reduce blood pressure (Serebrovskaya et al., 2008). Changes in heart rate variability (a useful marker of the autonomic nervous system activity, Malik, 1996) induced during and after hypoxic conditioning sessions remain to be elucidated. For example, it is known that after 12 h of sustained hypoxia (4000 m), a pronounced (dampened) effect on heart rate variability is evident, even 1 h after returning to normoxic ambient air (Guger et al., 2008), while after a brief hypoxic exposure of 15 min (FiO_2_ = 0.11), the hypoxia-induced reduction in heart rate variability disappears almost immediately (Roche et al., 2002). Hence, in order to provide potential physiological biomarkers for the implementation of hypoxic conditioning strategies, the effects of repetitive SH or IH exposure over several days need to be clarified especially on important cardiovascular mechanisms such as the autonomic nervous system.

Another issue to consider when applying hypoxic conditioning strategies is the determination of tissue-specific responses to hypoxic exposure which are generally characterized based on the magnitude of arterial blood deoxygenation. Important differences in oxygenation levels between tissues have been shown when FiO_2_ is reduced both in animal models (Almendros et al., 2011; Reinke et al., 2011) and in healthy subjects (Rupp et al., 2011, 2016). Rupp et al. (2016) demonstrated that healthy subjects exposed to intermittent hypoxemia for 45 min showed significant cyclical prefrontal cortex deoxygenation while the biceps brachialis muscle remained normoxic. Hence, changes in tissue (especially muscle and brain) oxygenation as measured non-invasively by near-infrared spectroscopy (NIRS) need to be characterized in order to better determine the organ-specific hypoxic stress applied during hypoxic conditioning sessions.

Therefore, the purpose of this study was to assess the acute and short-term cardiorespiratory and tissue oxygenation effects of two hypoxic conditioning strategies (i.e., sustained vs. intermittent hypoxic exposures as previously used in the literature) in order to determine the most efficient strategy and to identify physiological biomarkers able to provide early assessment of each hypoxic conditioning program, with emphasis on distinguishing protective vs. deleterious responses. We hypothesized that IH conditioning would induce larger cardiorespiratory (i.e., improved blood pressure and heart rate variability) benefits due to repetitive deoxygenation-reoxygenation cycles compared to SH conditioning.

## Materials and methods

### Subjects

Fourteen (7 females) active, healthy volunteers (age: 25 ± 8 years, body mass: 72 ± 11 kg, height: 179 ± 7 cm, body mass index 21.5 ± 2.5 kg·m^−2^) were included in the present study after routine medical assessment consisting of a short clinical examination, a 12-lead electrocardiogram and a respiratory function test. All subjects were non-smokers and had no history of cardiorespiratory or neuromuscular disease. They lived at sea level (Grenoble, 212 m) and were not exposed to altitude >1000 m over the past 3 months. Subjects were engaged 1–3 times a week in low- to moderate-intensity endurance activities such as walking, jogging, or cycling. They were instructed to keep their diet, hydration status, and physical activity routine identical throughout the study. Subjects refrained from physical exercise the days prior to the tests, abstained from drinking caffeinated beverages on test days, and had their last meal at least 2 h prior to the tests. The study was approved by the local ethics committee (CPP Sud-Est V) and performed according to the Declaration of Helsinki. Subjects were fully informed of the procedure and risks involved and gave their written consent prior to all assessments.

### Experimental design

In this prospective, randomized, cross-over, controlled, single-blind study performed in Grenoble (altitude: 210 m), all participants performed first a full familiarization trial in normoxia including sitting and breathing quietly through a facemask, with continuous electrocardiogram and blood pressure measurements. Then, they were randomized (using block randomization) into either the sustained hypoxia (SH) or intermittent hypoxia (IH) condition. After performing the first hypoxic condition (three ~1-h sessions separated by 48 h), all subjects completed the second hypoxic condition (three ~1-h sessions separated by 48 h) after a minimum washout period of 15 days. Cardiorespiratory and NIRS signals were continuously recorded on session 1 (S1), and session 3 (S3) of each hypoxic condition (described below). On session 2, only SpO_2_ and FiO_2_ were recorded. This study focused on physiological responses over three sessions of each hypoxic conditioning strategy since this represents a reasonable time frame (1 week) to assess the early effects of this intervention (Rodway et al., 2007) and to adapt, if required, the hypoxic dose.

**Figure 1:**
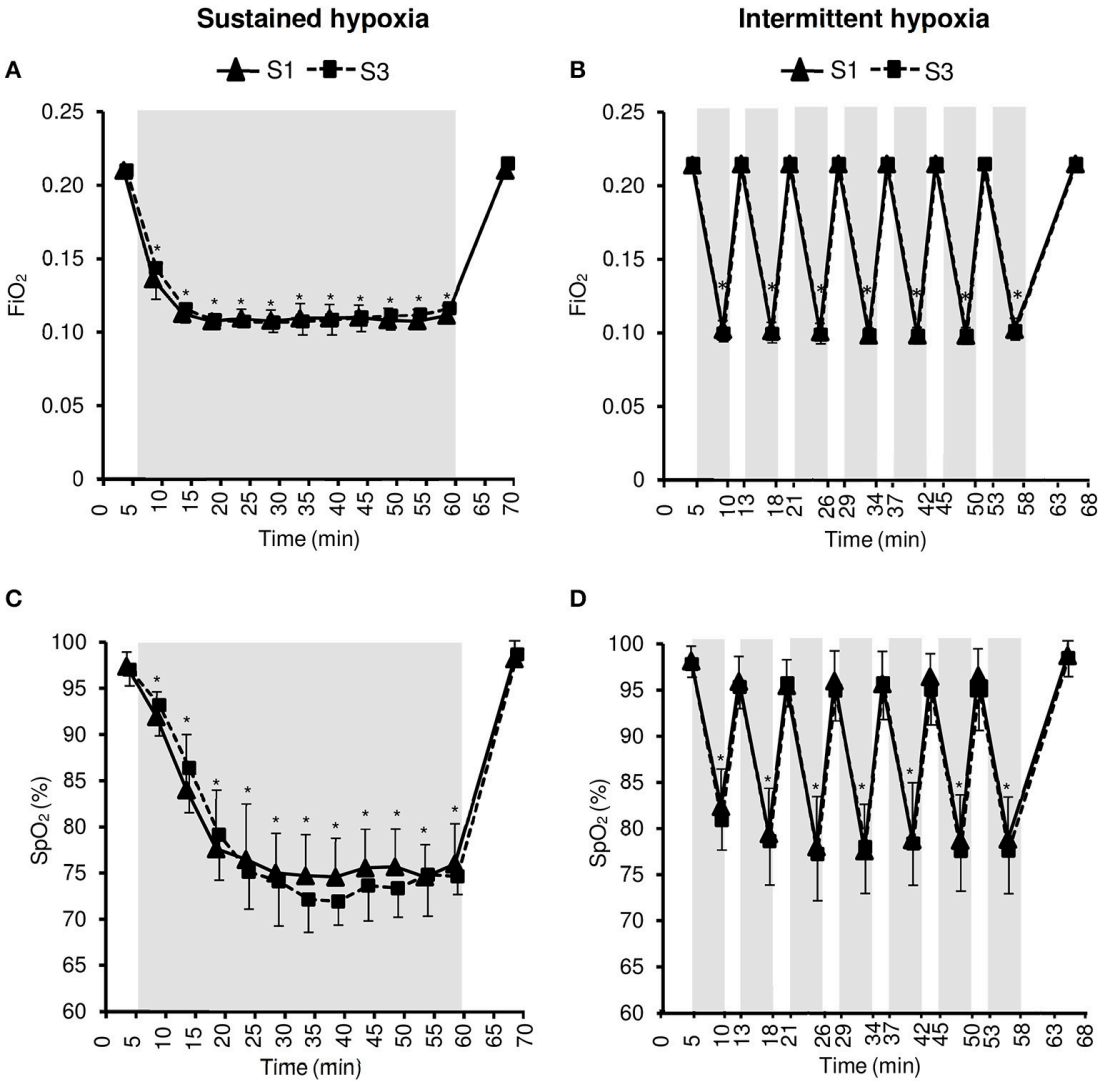
**Inspiratory oxygen fraction (FiO_2_, A,B)** and arterial oxygen saturation (SpO_2_, **C,D**) during the first (S1) and the third (S3) sustained or intermittent hypoxia sessions. Data points are means ± *SD*. Gray areas indicate hypoxic phases. ^*^Significantly different from normoxic baseline in both S1 and S3 (*p* < 0.05, two-way ANOVA main effect of time).

In all testing sessions subjects seated in a semi-supine position, in a quiet environment, at rest but without sleeping. They were blinded for the gas mixture composition they were inhaling. Subjects were told that this study aimed to compare the effect of two different types of hypoxic exposure, without further details. The hypoxic stimulus was obtained by having subjects breathe via a facemask a nitrogen-enriched gas mixture provided by a gas-mixing device (Altitrainer®, SMTEC S.A., Nyon Switzerland). FiO_2_ was individually adjusted to reach the targeted SpO_2_ of 70–80% in both hypoxic conditioning interventions (see Figure 1).

### Sustained hypoxia (SH) sessions

SH sessions started with an initial phase of 5 min breathing normoxic, ambient air for baseline data collection. Then, the hypoxic phase began with a progressive decrease of FiO_2_ from 0.21 to 0.10 over 10 min. Afterwards, FiO_2_ was automatically adjusted to reach 70–80% SpO_2_. This target SpO_2_ level was sustained until 55 min of hypoxic exposure. There was a final normoxic recovery phase where participants breathed ambient air for an additional 10 min, for a total testing duration of 70 min.

### Intermittent hypoxia (IH) sessions

IH sessions consisted of a 5 min baseline breathing normoxic, ambient air, followed by seven cycles of 5-min hypoxia (breathing a gas mixture with reduced FiO_2_ to reach the target SpO_2_of 70–80%) and 3-min normoxic breathing. The initial 5-min hypoxia was performed with a FiO_2_ of 0.10 for all subjects, and FiO_2_ was individually adjusted for the subsequent 5-min hypoxic phases in order to reach the target SpO_2_. After the seven hypoxia–normoxia cycles, 7 min of normoxic recovery were performed for a total session duration of 68 min.

### Cardiorespiratory measurements

Ventilation and gas exchanges were monitored continuously breath-by-breath using the MetaMax 3B (Cortex Biophysik GmbH, Leipzig, Germany). Gas analyzers and volume transducers were calibrated prior to each test with a 3-L syringe and references gases, respectively, according to manufacturer's instructions. Blood pressure was measured on the dominant arm twice at each time point with a digital pressure monitor system (A&D Medical, Kitamoto-Shi, Japan). ECG was continuously recorded using a 2-lead ECG at a sampling rate of 1000 Hz (Holter AFT 1000, Holter supplies, Paris, France).

### NIRS measurements

Oxy[HbO2]-, deoxy[HHb]-, and total[HbTot]-hemoglobin concentration changes and tissue oxygenation index (TSI) were estimated continuously throughout testing sessions over multiple sites using a two-wavelength (780 and 850 nm) multichannel, continuous wave NIRS system (Oxymon MkIII, Artinis Medical Systems, Netherlands). Muscle hemodynamics were assessed at the middle of right *biceps brachialis* using a 4-cm interoptode distance. Left pre-frontal cortex hemodynamics were assessed between Fp1 and F3 locations according to the international 10–20 EEG system with 3.5-cm inter-optode distance. The probe holders were secured to the skin with double-sided tape and maintained with Velcro bands. Data were recorded continuously at 10 Hz and filtered with a 2-s width moving Gaussian smoothing algorithm before analysis.

### Data analysis

ECG data was transferred to the QuickReader software (Holter supplies) and R–R intervals were analyzed by means of the Kubios HRV Analysis Software 2.2 (Tarvainen et al., 2002, 2014). Heart rate variability parameters were recorded and calculated in accordance with the task force of the European society of cardiology and the North American society of pacing and electrophysiology (Malik, 1996). Data were visually inspected to remove artifacts and heart rate variability parameters were calculated over 5-min periods. The following parameters were used to analyze the heart rate variability within the time domain: standard deviation of R–R interval (SDNN), percentage of successive R–R that varied by 50 ms or more (PNN50) and the root-mean-square difference of successive normal R–R intervals (RMSSD). In the frequency domain, the following parameters were calculated using a Fast Fourier Transform: low frequency (LF; 0.04–0.15 Hz), high frequency (HF; 0.15–0.40 Hz), and LF/HF ratio.

During SH sessions, minute ventilation (V_E_), gas exchanges, heart rate, SpO_2_, and NIRS parameters were averaged over the last 30 s of the baseline normoxic phase, of each 5-min periods in hypoxia and of the normoxic recovery phase. During IH sessions, the same parameters were averaged over the last 30 s of the baseline normoxic phase, of each 5-min hypoxic, and 3-min normoxic periods, and of the normoxic recovery phase. Arterial blood pressure was calculated as the average of two measurements at each of these time points. Heart rate variability parameters were calculated over the 5-min baseline normoxic phase, every 5 min during the hypoxic phase of SH sessions and during every 5-min hypoxic phases of IH sessions, and over the last 5 min of the normoxic recovery phase.

### Statistical analysis

All statistical procedures were completed on Statistica version 10 (Statsoft, Tulsa, OK). Normality of distribution and homogeneity of variances of the main variables were confirmed using a Shapiro-Wilk normality test and the Levene's test, respectively. For each hypoxic condition (SH and IH), in order to assess changes over time within the sessions and changes from S1 to S3, a two-way ANOVA (time × session) with repeated measures was performed for each dependent variable (see statistical results provided in figures). In order to assess differences between hypoxic conditions (SH vs. IH), a three-way ANOVA (time × session × condition) with repeated measures was performed for each dependent variable, using four time points only [baseline normoxia, min 25 (mid-session) and 60 (end-session) in SH and min 26 (mid-session) and 58 (end-session) in IH, and normoxic recovery). These four time points were selected in order to compare SH and IH under similar hypoxemic conditions, i.e., at mid and end hypoxic phase, when SpO_2_ remained at the same 70–80% target value for at least 5 min in both SH and IH (see statistical results provided in tables). *Post-hoc* Tukey's tests were applied to determine a difference between two mean values if the ANOVA revealed a significant main effect or interaction effect. For all statistical analyses, a two-tailed alpha level of 0.05 was used as the cut-off for significance. All data are presented as mean values ± *SD* within text and tables.

## Results

Subjects reported no significant discomfort and no adverse effect was observed during all SH and IH sessions.

### Inspiratory oxygen fraction and arterial oxygenation

Changes in FiO_2_ and SpO_2_ during the hypoxic conditioning sessions are presented in Figure 1 and Table 1. FiO_2_ was significantly reduced from baseline normoxic values throughout SH sessions (two-way ANOVA main effect of time *F* = 807.5, *p* < 0.001) and during the hypoxic phases of IH sessions (two-way ANOVA main effect of time *F* = 4762.0, *p* < 0.001). FiO_2_ reduction magnitude was similar between S1 and S3 in both conditions (SH two-way ANOVA, main effect of session *F* = 1.4, *p* = 0.262; IH two-way ANOVA main effect of session *F* = 1.0, *p* = 0.340) and was significantly larger at mid- and end-session in IH vs. SH (three-way ANOVA condition × time interaction *F* = 30.2, *p* < 0.001; Table 1). SpO_2_ was reduced from baseline normoxic values throughout SH sessions (two-way ANOVA main effect of time *F* = 171.5, *p* < 0.001) and during the hypoxic phases of IH sessions (two-way ANOVA main effect of time *F* = 213.7, *p* < 0.001). SpO_2_ reduction (SH 77.5 ± 1.7 and IH 78.2 ± 3.2%, on average during the hypoxic phases) was similar between S1 and S3 in both conditions (SH two-way ANOVA main effect of session *F* = 0.5, *p* = 0.482; IH two-way ANOVA main effect of session *F* = 1.7, *p* = 0.211) and was significantly larger at the end of the session in SH vs. IH (three-way ANOVA condition × time interaction *F* = 3.1, *p* = 0.040; Table 1).

**Table 1:** **Inspiratory oxygen fraction, arterial oxygen saturation, ventilation and cardiovascular responses during the first and the third sustained or intermittent hypoxia sessions**.

		**Sustained hypoxia**				**Intermittent hypoxia**				**3-way ANOVA**		
	**Session**	**Baseline** **normoxia**	**Middle** **hypoxia**	**End** **hypoxia**	**Recovery** **normoxia**	**Baseline** **normoxia**	**Middle** **hypoxia**	**End** **hypoxia**	**Recovery** **normoxia**	**Condition** **main effect**	**Condition x** **time interaction**	**Condition x** **session interaction**
FiO_2_	S1	0.21 (0.00)	0.11 (0.01)	0.11 (0.01)	0.21 (0.00)	0.21 (0.00)	0.10 (0.01)	0.10 (0.01)	0.21 (0.00)	*p* = 0.002	*p* < 0.001	*p* = 0.114
	S3	0.21 (0.00)	0.11 (0.00)	0.12 (0.01)	0.21 (0.00)	0.21 (0.00)	0.10 (0.01)	0.10 (0.01)	0.21 (0.00)			
SpO_2_ (%)	S1	97.4 (1.6)	76.5 (6.0)	75.9 (4.4)	98.1 (2.5)	98.1 (1.6)	77.9 (5.5)	78.8 (4.6)	98.7 (1.6)	*p* = 0.008	*p* = 0.040	*p* = 0.939
	S3	97.0 (1.8)	75.1 (4.0)	74.6 (1.9)	98.7 (1.3)	97.8 (1.4)	77.2 (5.0)	77.6 (4.6)	98.4 (2.0)			
PetCO_2_ (mmHg)	S1	37.5 (2.7)	35.0 (2.1)	34.2 (2.7)	36.7 (2.5)	37.2 (2.8)	34.0 (3.1)	34.0 (2.5)	35.9 (3.4)	*p* = 0.131	*p* = 0.612	*p* = 0.581
	S3	37.5 (2.2)	35.8 (2.1)	35.5 (3.0)	36.8 (3.5)	35.9 (2.4)	34.2 (1.9)	34.4 (2.7)	36.4 (2.0)			
V_E_ (L·min^−1^)	S1	9.7 (2.7)	10.7 (3.4)	10.7 (3.1)	9.0 (2.6)	10.5 (2.3)	11.8 (2.1)	11.3 (3.2)	9.3 (2.8)	*p* = 0.428	*p* = 0.617	*p* = 0.451
	S3	11.0 (3.6)	11.2 (3.9)	11.2 (4.0)	9.8 (2.9)	11.2 (2.1)	11.1 (2.5)	11.7 (3.9)	9.7 (2.6)			
HR (bpm)	S1	65 (15)	73 (15)	74 (12)	61 (12)	61 (8)	71 (11)	73 (10)	60 (10)	*p* = 0.251	*p* = 0.564	*p* = 0.834
	S3	64 (8)	76 (11)	74 (10)	60 (10)	63 (6)	74 (10)	73 (8)	59 (10)			
SBP (mmHg)	S1	115 (14)	119 (15)	120 (16)	125 (18)	113 (14)	115 (14)	118 (13)	116 (11)	*p* = 0.195	*p* = 0.899	*p* = 0.633
	S3	110 (13)	119 (18)	119 (18)	120 (16)	105 (14)	114 (16)	114 (13)	116 (14)			
DBP (mmHg)	S1	67 (11)	68 (9)	72 (7)	75 (8)	63 (12)	64 (12)	68 (10)	68 (9)	*p* = 0.295	*p* = 0.779	*p* = 0.143
	S3	62 (12)	65 (16)	69 (10)	70 (10)	62 (12)	65 (10)	69 (9)	70 (9)			

*Values are provided for the normoxic phases before and after hypoxic exposure and in the middle and the end of the hypoxic exposure. Data are means (SD); FiO_2_, inspiratory oxygen fraction; SpO_2_, arterial oxygen saturation; PetCO_2_, end tidal carbon dioxide partial pressure; V_E_, minute ventilation; HR, heart rate; SBP, systolic blood pressure; DBP, diastolic blood pressure; S1, first session; S3, third session*.

### Cardiorespiratory responses

**Figure 2:**
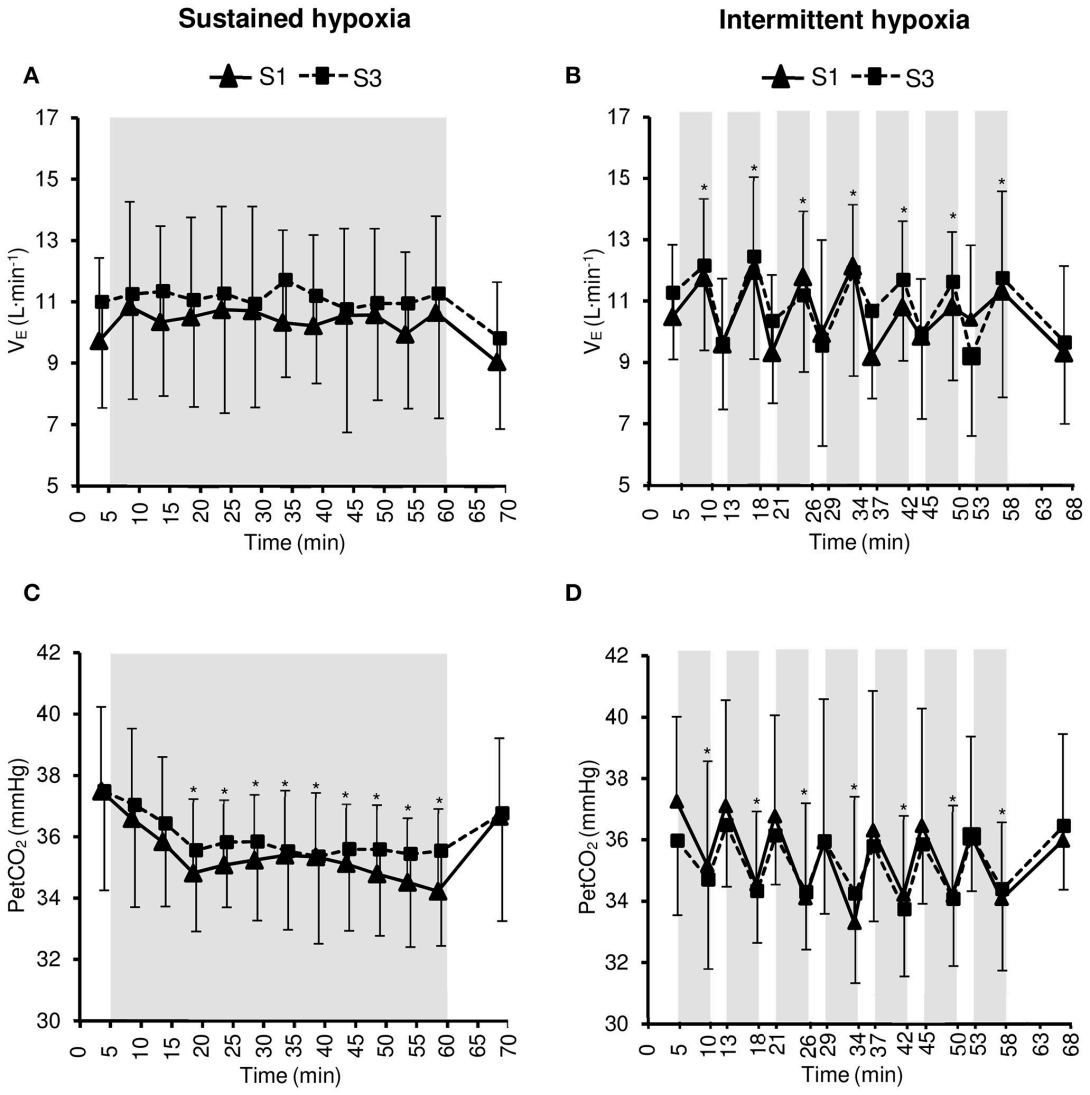
**Minute ventilation (V_E_, A,B)** and end-tidal CO_2_ partial pressure (PetCO_2_, **C,D**) during the first (S1) and the third (S3) sustained or intermittent hypoxia sessions. Data points are means ± *SD*. Gray areas indicate hypoxic phases. ^*^Significantly different from normoxic baseline in both S1 and S3 (*p* < 0.05, two-way ANOVA main effect of time).

Changes in V_*E*_ and PetCO_2_ during the hypoxic conditioning sessions are presented in Figure 2 and Table 1. A significant increase in V_E_ from baseline normoxic values was observed during the hypoxic phases of IH sessions both in S1 and S3 (two-way ANOVA main effect of time *F* = 6.1, *p* < 0.001) but not during SH sessions (two-way ANOVA main effect of time *F* = 1.6, *p* = 0.108). PetCO_2_ decreased significantly from baseline normoxic values during SH sessions both in S1 and S3 (two-way ANOVA main effect of time *F* = 9.0, *p* < 0.001) and during the hypoxic phases of IH sessions both in S1 and S3 (two-way ANOVA main effect of time *F* = 10.2, *p* < 0.001). V_E_ and PetCO_2_ changes did not differ between SH and IH at mid- and end-session (V_E_ three-way ANOVA condition × time interaction *F* = 0.6, *p* = 0.617; PetCO_2_ three-way ANOVA condition × time interaction *F* = 0.6, *p* = 0.612; Table 1).

**Figure 3:**
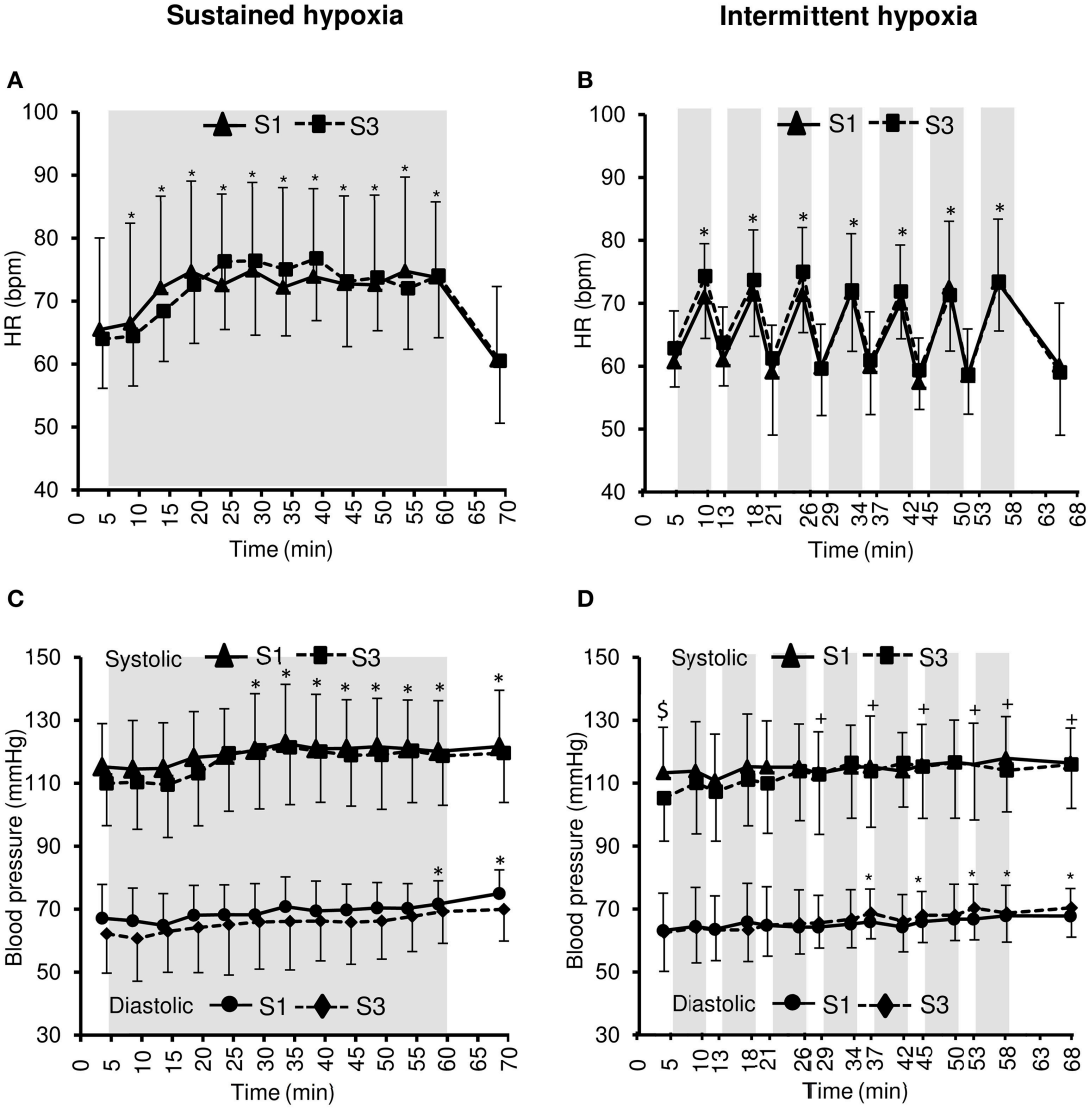
**Heart rate (HR, A,B)** and systolic and diastolic blood pressure **(C,D)** during the first (S1) and the third (S3) sustained or intermittent hypoxia sessions. Data points are means ± *SD*. Gray areas indicate hypoxic phases. ^*^Significantly different from normoxic baseline in both S1 and S3 (*p* < 0.05, two-way ANOVA main effect of time); ^+^Significantly different from normoxic baseline in S3 only (*p* < 0.05, two-way ANOVA session × time interaction); ^*$*^Significant difference between S1 and S3 (*p* < 0.05, two-way ANOVA session × time interaction).

Changes in heart rate and arterial blood pressure during the hypoxic conditioning sessions are presented in Figure 3 and Table 1. A significant increase in heart rate from baseline normoxic values was observed throughout SH sessions (two-way ANOVA main effect of time *F* = 30.0, *p* < 0.001) and during the hypoxic phases of IH sessions (two-way ANOVA main effect of time *F* = 57.5, *p* < 0.001) both in S1 and S3. Heart rate increase was similar in SH and IH (three-way ANOVA main condition effect *F* = 1.4, *p* = 0.251; Table 1). Systolic blood pressure (SBP) increased significantly from baseline normoxic values during SH both in S1 and S3 (two-way ANOVA main effect of time *F* = 21.0, *p* < 0.001) while it increased during IH in S3 only (two-way ANOVA session × time interaction *F* = 2.1, *p* = 0.013). Baseline normoxic SBP was significantly reduced at S3 compared to S1 in IH (*p* = 0.009). Diastolic blood pressure (DBP) increased significantly from baseline normoxic values during SH and IH both in S1 and S3 (SH two-way ANOVA main effect of time *F* = 5.5, *p* < 0.001; IH two-way ANOVA main effect of time *F* = 6.5, *p* < 0.001). Changes in SBP and DBP did not differ between SH and IH conditions (Table 1).

### Heart rate variability

**Figure 4:**
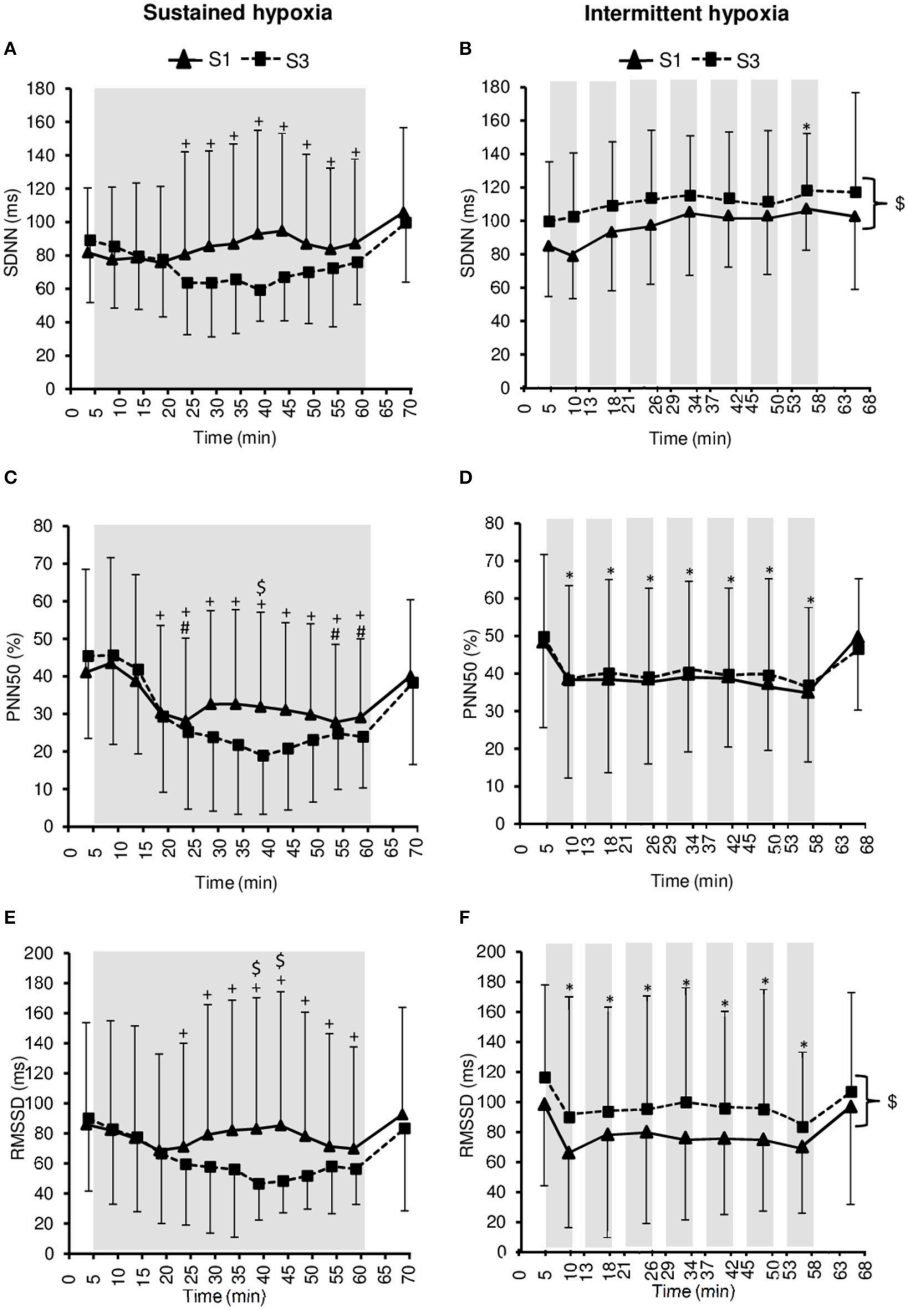
**Heart rate variability indices within the time domain [standard deviation of R–R interval (SDNN), (A,B)**; percentage of successive R–R that varied by 50 ms or more (PNN50), **(C,D)**; root-mean-square difference of successive normal R–R intervals (RMSSD), **(E,F)]** during the first (S1) and the third (S3) sustained or intermittent hypoxia sessions. Data points are means ± *SD*. Gray areas indicate hypoxic phases. ^*^Significantly different from normoxic baseline in both S1 and S3 (*p* < 0.05, two-way ANOVA main effect of time); ^+^Significantly different from normoxic baseline in S3 only (*p* < 0.05, two-way ANOVA session × time interaction); ^#^Significantly different from normoxic baseline in S1 only (*p* < 0.05, two-way ANOVA session × time interaction); ^*$*^Significant difference between S1 and S3 (*p* < 0.05, two-way ANOVA main effect of session or session × time interaction).

**Figure 5:**
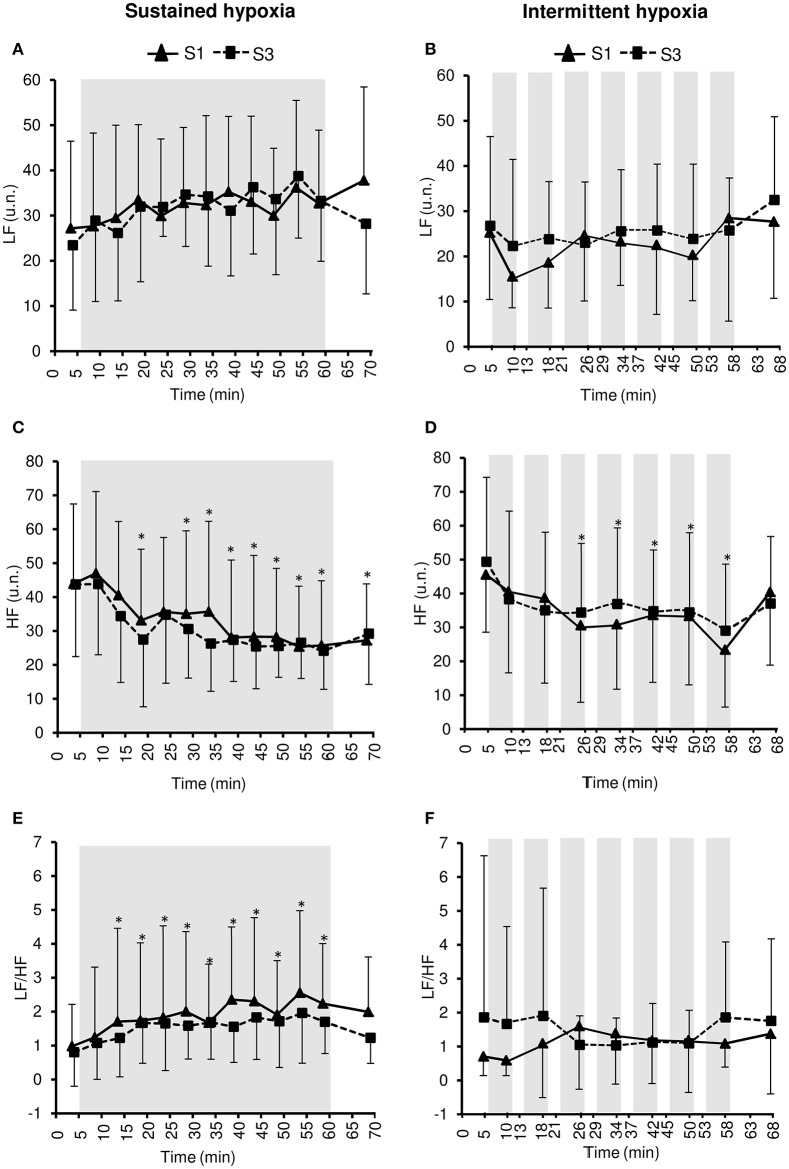
**Heart rate variability indices within the frequency domain [low frequency in normalized units (LF), (A,B)**; high frequency in normalized units (HF), **(C,D)**; LF/HF ratio, **(E,F)**] during the first (S1) and the third (S3) sustained or intermittent hypoxia sessions. Data points are means ± *SD*. Gray areas indicate hypoxic phases. ^*^Significantly different from normoxic baseline in both S1 and S3 (*p* < 0.05, two-way ANOVA main effect of time).

Changes in heart rate variability parameters during the hypoxic conditioning sessions are presented in Figures 4, 5 and Table 2. SDNN decreased significantly in SH during S3 (two-way ANOVA condition × time interaction *F* = 2.9, *p* = 0.001) and increased significantly in IH during both S1 and S3 (two-way ANOVA main effect of time *F* = 3.2, *p* = 0.003). SDNN in IH was significantly larger during S3 compared to S1 (two-way ANOVA main effect of session *F* = 4.9, *p* = 0.045). SDNN was significantly lower in SH compared to IH (three-way ANOVA main effect of condition *F* = 23.4, *p* < 0.001; three-way ANOVA condition × time interaction *F* = 5.8, *p* = 0.002; Table 2). PNN50 decreased during both S1 and S3 in SH (two-way ANOVA main effect of time *F* = 10.1, *p* < 0.001) and IH (two-way ANOVA main effect of time *F* = 5.9, *p* < 0.001) sessions. PNN50 in SH decreased to a greater extent during S3 compared to S1 (two-way ANOVA condition × time interaction *F* = 5.8, *p* = 0.002). PNN50 was significantly lower in SH compared to IH (three-way ANOVA main effect of condition *F* = 8.7, *p* = 0.011; Table 2). RMSSD decreased during S3 in SH (two-way ANOVA session × time interaction *F* = 3.3, *p* < 0.001) and during both S1 and S3 in IH (two-way ANOVA main effect of time *F* = 4.6, *p* < 0.001) sessions. RMSSD in SH decreased to a greater extent during S3 compared to S1 (two-way ANOVA session × time interaction *F* = 3.3, *p* < 0.001) while in IH it was larger during S3 compared to S1 (two-way ANOVA main effect of session *F* = 4.2, *p* = 0.046). RMSSD was significantly lower in SH compared to IH (three-way ANOVA main effect of condition *F* = 8.9, *p* = 0.010; Table 2).

**Table 2:** **Heart rate variability indices during the first and the third sustained or intermittent hypoxia sessions**.

		**Sustained hypoxia**				**Intermittent hypoxia**				**ANOVA**		
	**Session**	**Baseline** **normoxia**	**Middle** **hypoxia**	**End** **hypoxia**	**Recovery** **normoxia**	**Baseline** **normoxia**	**Middle** **hypoxia**	**End** **hypoxia**	**Recovery** **normoxia**	**Condition** **main effect**	**Condition x** **time interaction**	**Condition x** **session interaction**
SDNN (ms)	S1	81.7 (38.6)	80.5 (61.4)	87.7 (50.6)	105.7 (50.8)	84.9 (30.3)	97.5 (35.5)	107.2 (24.7)	102.6 (43.7)	*p* < 0.001	*p* = 0.002	*p* = 0.100
	S3	89.0 (37.1)	63.5 (31.0)	75.8 (25.2)	99.5 (35.5)	99.6 (35.7)	113.5 (40.6)	118.1 (34.1)	117.0 (59.6)			
PNN50 (%)	S1	41.0 (27.4)	28.1 (22.0)	29.0 (20.9)	40.0 (20.3)	48.4 (22.7)	38.6 (22.6)	35.0 (18.5)	49.7 (19.4)	*p* = 0.011	*p* = 0.362	*p* = 0.783
	S3	45.0 (21.9)	25.2 (20.5)	23.9 (13.5)	38.3 (21.8)	49.7 (21.9)	39.0 (23.6)	36.8 (20.7)	46.4 (18.7)			
RMSSD (ms)	S1	85.8 (67.7)	70.9 (69.0)	69.6 (67.9)	92.5 (71.3)	98.5 (54.2)	80.2 (60.9)	70.4 (44.4)	97.03 (61.5)	*p* = 0.010	*p* = 0.638	*p* = 0.156
	S3	90.0 (48.3)	59.3 (40.2)	56.2 (23.5)	83.3 (54.8)	116.3 (61.5)	95.3 (75.2)	83.3 (49.7)	106.7 (66.1)			
LF (u.n.)	S1	27.1 (19.2)	29.8 (17.0)	32.6 (16.1)	37.7 (20.6)	25.2 (14.7)	24.6 (14.5)	28.4 (22.8)	27.6 (16.9)	*p* = 0.167	*p* = 0.115	*p* = 0.478
	S3	23.4 (14.3)	31.8 (6.4)	33.2 (13.3)	28.1 (15.4)	26.7 (19.7)	22.9 (13.4)	25.7 (11.6)	32.4 (18.4)			
HF (u.n.)	S1	43.9 (23.5)	35.6 (21.9)	25.7 (19.0)	27.2 (16.6)	45.5 (16.9)	30.61 (22.7)	23.4 (16.9)	40.4 (21.5)	*p* = 0.180	*p* = 0.123	*p* = 0.407
	S3	43.7 (21.2)	34.7 (20.1)	24.1 (11.3)	29.1 (14.9)	49.3 (24.9)	34.3 (20.4)	29.0 (19.6)	37.0 (19.7)			
LF/HF	S1	0.97 (1.23)	1.82 (2.70)	2.23 (1.77)	1.99 (1.62)	0.70 (0.56)	1.59 (1.86)	1.08 (0.69)	1.37 (1.77)	*p* = 0.658	*p* = 0.231	*p* = 0.239
	S3	0.80 (0.77)	1.65 (1.38)	1.69 (0.92)	1.22 (0.74)	1.85 (4.77)	1.04 (0.86)	1.85 (2.23)	1.75 (2.42)			

*Values are provided for the normoxic phases before and after hypoxic exposure and in the middle and the end of the hypoxic exposure. Data are means (SD); SDNN, standard deviation of R–R interval; PNN50, percentage of successive R–R that varied by 50 ms or more; RMSSD, root-mean-square difference of successive normal R–R intervals; LF, low frequency in normalized units; HF, high frequency in normalized units; S1, first session; S3, third session*.

LF did not change during both S1 and S3 in SH (two-way ANOVA main effect of time *F* = 1.8, *p* = 0.054) and IH (two-way ANOVA main effect of time *F* = 1.4, *p* = 0.207). HF decreased significantly during both S1 and S3 in SH (two-way ANOVA main effect of time *F* = 8.4, *p* < 0.001) and IH (two-way ANOVA main effect of time *F* = 6.6, *p* < 0.001) sessions. LF/HF increased significantly during both S1 and S3 in SH (two-way ANOVA main effect of time *F* = 4.6, *p* < 0.001) but not in IH (two-way ANOVA main effect of time *F* = 0.5, *p* = 0.868). LF/HF did not differ significantly between SH and IH (three-way ANOVA main effect of condition *F* = 0.2, *p* = 0.658; three-way ANOVA condition × time interaction *F* = 1.5, *p* = 0.231; Table 2).

### Tissue oxygenation

**Figure 6:**
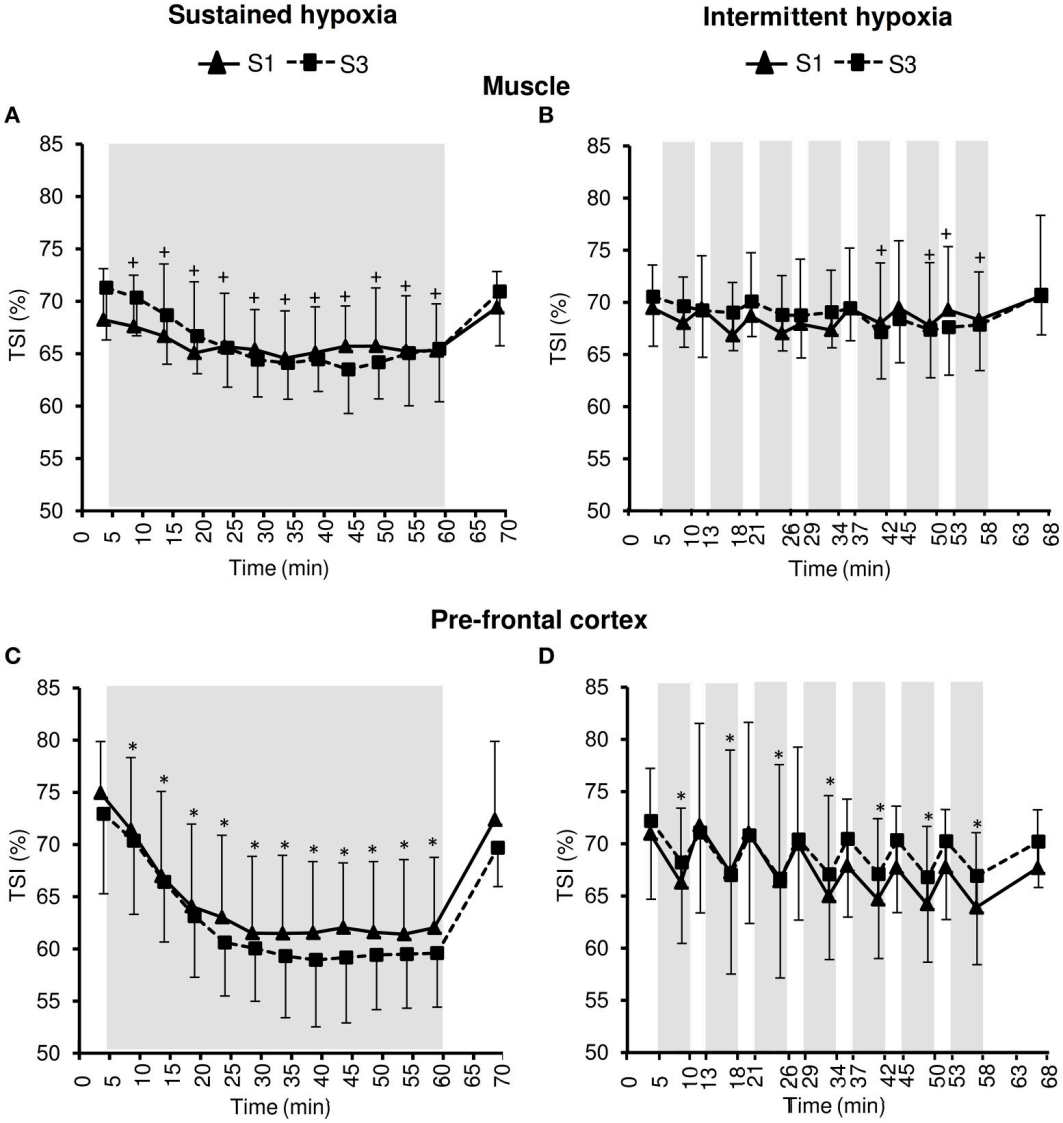
**Tissue oxygenation index (TSI) for the muscle (A,B)** and the prefrontal cortex **(C,D)** during the first (S1) and the third (S3) sustained or intermittent hypoxia sessions. Data points are means ± *SD*. Gray areas indicate hypoxic phases. ^*^Significantly different from normoxic baseline in both S1 and S3 (*p* < 0.05, two-way ANOVA main effect of time); ^+^Significantly different from normoxic baseline in S3 only (*p* < 0.05, two-way ANOVA session × time interaction).

**Figure 7:**
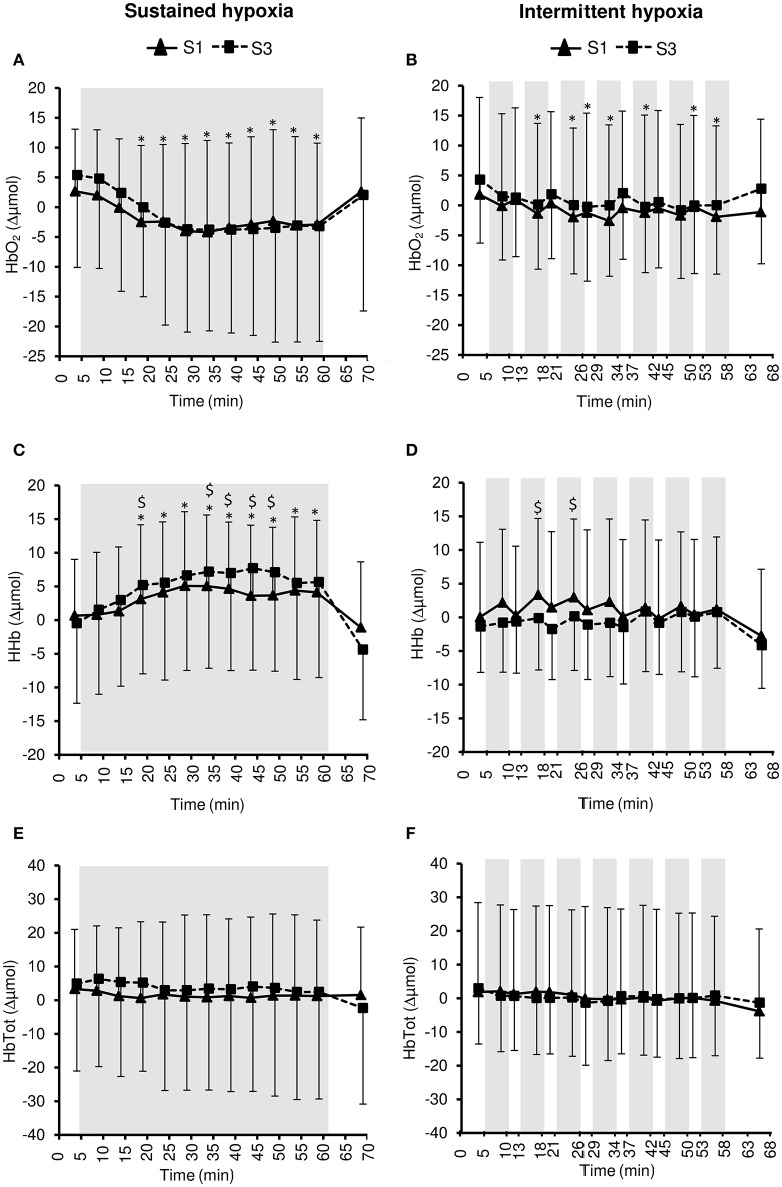
**Muscle oxy- ([HbO2], A,B)**, deoxy- ([HHb], **C,D**), and total- ([HbTot], **E,F**) hemoglobin concentration changes during the first (S1) and the third (S3) sustained or intermittent hypoxia sessions. Data points are means ± SD. Gray areas indicate hypoxic phases. ^*^Significantly different from normoxic baseline in both S1 and S3 (*p* < 0.05, two-way ANOVA main effect of time); ^*$*^Significant difference between S1 and S3 (*p* < 0.05, two-way ANOVA session × time interaction).

Changes in muscle NIRS parameters during the hypoxic conditioning sessions are presented in Figures 6, 7 and Table 3. Muscle TSI decreased significantly in S3 but not in S1 during both SH (two-way ANOVA session × time interaction *F* = 3.1, *p* = 0.001) and IH (two-way ANOVA session × time interaction *F* = 2.3, *p* = 0.006) sessions. Muscle TSI reduction was similar between SH and IH conditions (three-way ANOVA condition × time interaction *F* = 2.2, *p* = 0.109; Table 3). Muscle HHb (SH two-way ANOVA session × time interaction *F* = 3.1, *p* = 0.001; IH two-way ANOVA session × time interaction *F* = 2.3, *p* = 0.007) but not HbO_2_ (SH two-way ANOVA session × time interaction *F* = 1.7, *p* = 0.077; IH two-way ANOVA session × time interaction *F* = 1.1, *p* = 0.395) nor HbTot (SH two-way ANOVA session × time interaction *F* = 1.8, *p* = 0.052; IH two-way ANOVA session × time interaction *F* = 1.2, *p* = 0.311) showed larger changes in S3 compared to S1 in both SH and IH conditions. HbO_2_ reduction (three-way ANOVA condition × time interaction *F* = 3.1, *p* = 0.037) and HHb increase (three-way ANOVA condition × time interaction *F* = 3.8, *p* = 0.017) were larger during SH compared to IH conditions (Table 3).

**Table 3:** **Muscle and pre-frontal cortex oxygenation during the first and the third sustained or intermittent hypoxia sessions**.

		**Sustained hypoxia**				**Intermittent hypoxia**				**ANOVA**		
	**Session**	**Baseline** **normoxia**	**Middle** **hypoxia**	**End** **hypoxia**	**Recovery** **normoxia**	**Baseline** **normoxia**	**Middle** **hypoxia**	**End** **hypoxia**	**Recovery** **normoxia**	**Condition** **main effect**	**Condition x** **time interaction**	**Condition x** **session interaction**
TSIm (%)	S1	68.2 (4.8)	65.6 (5.0)	65.2 (4.4)	69.4 (3.4)	69.4 (4.1)	67.0 (5.5)	68.3 (4.5)	70.6 (7.7)	*p* = 0.130	*p* = 0.109	*p* = 0.582
	S3	71.2 (4.9)	65.5 (3.7)	65.4 (5.0)	70.9 (5.1)	70.5 (4.7)	68.7 (3.4)	67.8 (4.3)	70.7 (3.8)			
HbO_2_m (Δμmol)	S1	2.6 (10.4)	−2.4 (12.9)	−2.9 (13.6)	2.6 (12.3)	1.8 (16.2)	−1.9 (14.8)	−1.9 (15.1)	−1.1 (15.5)	*p* = 0.280	*p* = 0.037	*p* = 0.195
	S3	5.3 (15.4)	−2.6 (17.1)	−3.1 (19.3)	2.0 (19.4)	4.2 (10.5)	0.0 (11.4)	0.0 (11.5)	2.7 (12.5)			
HHbm (Δμmol)	S1	0.6 (8.3)	4.1 (10.4)	4.1 (10.6)	−1.0 (9.7)	0.0 (11.1)	2.9 (11.6)	1.1 (10.7)	−2.7 (9.8)	*p* = 0.160	*p* = 0.017	*p* = 0.247
	S3	−0.4 (11.8)	5.5 (14.4)	5.6 (14.1)	−4.3 (10.4)	−1.3 (6.8)	0.1 (8.0)	0.7 (8.3)	−4.1 (6.4)			
HbTotm (Δμmol)	S1	3.3 (17.6)	1.6 (21.5)	1.2 (22.5)	1.5 (20.1)	1.8 (26.5)	1.0 (25.2)	−0.7 (25.0)	−3.8 (24.4)	*p* = 0.967	*p* = 0.995	*p* = 0.741
	S3	4.9 (25.9)	2.9 (29.7)	2.4 (31.8)	−2.3 (28.4)	2.9 (16.4)	0.1 (17.4)	0.8 (17.8)	−1.3 (16.4)			
TSIc (%)	S1	74.9 (4.9)	63.0 (7.8)	62.0 (6.7)	72.3 (7.5)	70.9 (6.2)	66.4 (11.1)	63.8 (7.1)	67.6 (5.5)	*p* = 0.213	*p* < 0.001	*p* = 0.128
	S3	72.9 (7.6)	60.6 (5.1)	59.5 (5.1)	69.6 (3.7)	72.1 (7.4)	66.6 (9.5)	66.9 (8.4)	70.2 (4.3)			
HbO_2_c (Δμmol)	S1	−2.3 (12.0)	−10.6 (12.3)	−12.3 (11.0)	−4.7 (11.9)	1.95 (11.7)	−5.6 (13.3)	−4.4 (13.9)	−2.7 (10.1)	*p* = 0.023	*p* < 0.001	*p* = 0.249
	S3	−3.4 (12.1)	−14.1 (10.0)	−15.1 (11.3)	−7.3 (11.0)	−1.8 (11.3)	−7.2 (11.0)	−7.8 (11.3)	−4.3 (11.0)			
HHbc (Δμmol)	S1	−1.2 (10.5)	8.5 (10.5)	10.2 (12.7)	1.2 (11.5)	1.8 (13.1)	8.1 (13.4)	7.9 (12.7)	0.2 (4.8)	*p* = 0.001	*p* < 0.001	*p* = 0.111
	S3	−0.9 (12.5)	9.5 (14.2)	11.1 (13.2)	−1.4 (7.6)	−0.1 (13.1)	5.3 (13.7)	5.3 (13.2)	−2.1 (6.1)			
HbTotc (Δμmol)	S1	−3.6 (22.4)	−2.0 (22.1)	−2.1 (22.0)	−3.5 (21)	3.8 (22.9)	2.5 (22.0)	3.5 (22.1)	−2.4 (12.5)	*p* = 0.200	*p* = 0.580	*p* = 0.485
	S3	−4.3 (23.3)	−4.6 (22.9)	−4.0 (23.1)	−8.7 (17.1)	−2.02 (22.4)	−1.9 (21.5)	−2.5 (20.3)	−6.4 (16.7)			

*Values are provided for the normoxic phases before and after hypoxic exposure and in the middle and the end of the hypoxic exposure. Data are means (SD); TSI, tissue saturation index; HbO_2_, oxyhemoglobin concentration; HHb, deoxyhemoglobin concentration; HbTot, total hemoglobin concentration; -m, muscle; -c; prefrontal cortex; S1, first session; S3, third session*.

**Figure 8:**
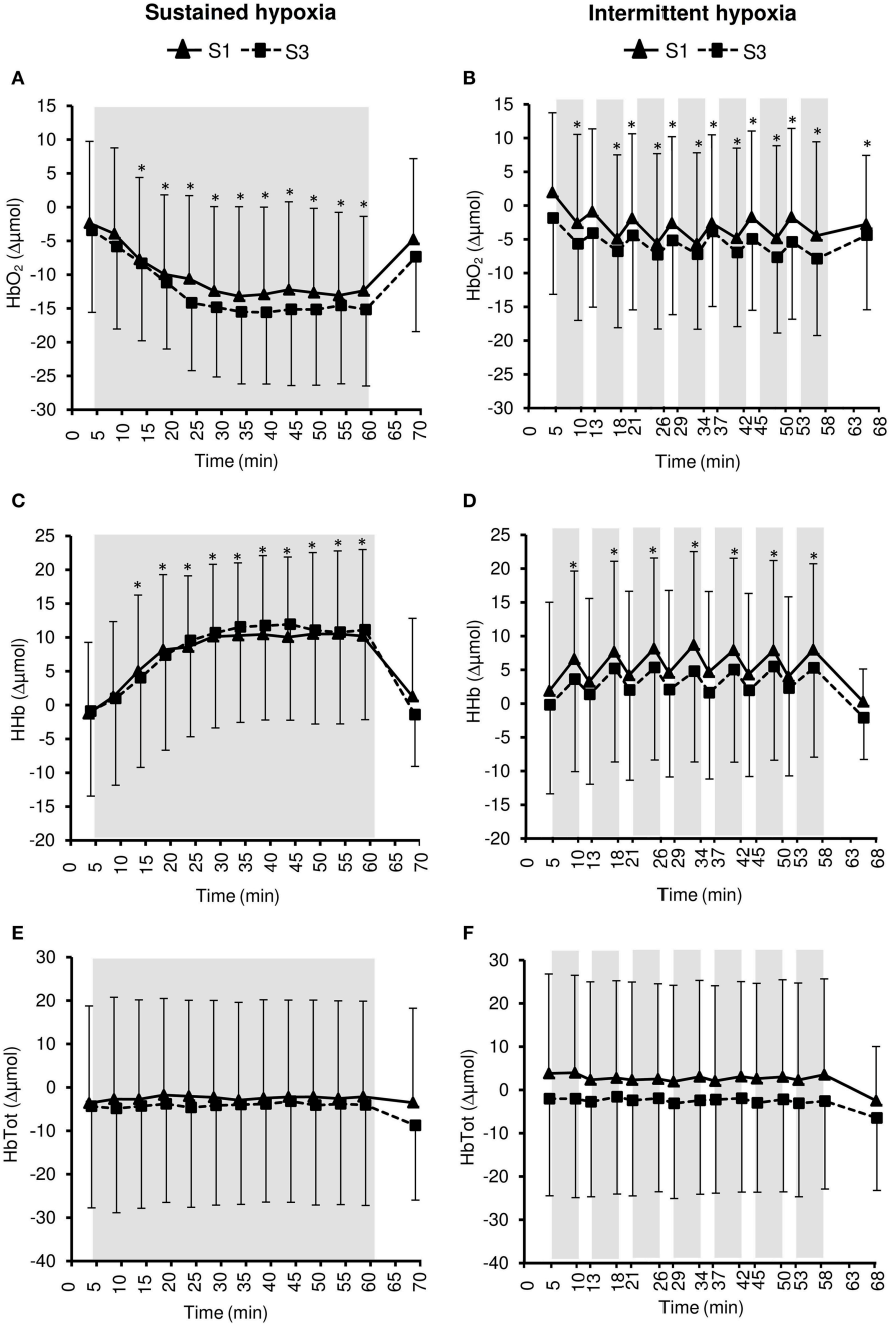
**Pre-frontal cortex oxy- ([HbO2], A,B)**, deoxy- ([HHb], **C,D**), and total- ([HbTot], **E,F**) hemoglobin concentration changes during the first (S1) and the third (S3) sustained or intermittent hypoxia sessions. Data points are means ± SD. Gray areas indicate hypoxic phases. ^*^Significantly different from normoxic baseline in both S1 and S3 (*p* < 0.05, two-way ANOVA main effect of time).

Changes in pre-frontal cortex NIRS parameters during the hypoxic conditioning sessions are presented in Figures 6, 8 and Table 3. Pre-frontal cortex TSI decreased significantly during both S1 and S3 in SH (two-way ANOVA main effect of time *F* = 75.4, *p* < 0.001) and IH (two-way ANOVA main effect of time *F* = 9.8, *p* < 0.001) sessions. Pre-frontal cortex HbO_2_ significantly decreased (SH two-way ANOVA main effect of time *F* = 36.7, *p* < 0.001; IH two-way ANOVA main effect of time *F* = 12.7, *p* < 0.001) whereas HHb significantly increased (SH two-way ANOVA main effect of time *F* = 98.7, *p* < 0.001; IH two-way ANOVA main effect of time *F* = 16.4, *p* < 0.001) during both S1 and S3 in SH and IH. Pre-frontal cortex HbTot did not change over time both in SH (two-way ANOVA main effect of time *F* = 0.9, *p* = 0.519) and IH (two-way ANOVA main effect of time *F* = 1.7, *p* = 0.056). Pre-frontal cortex TSI (three-way ANOVA condition × time interaction *F* = 15.4, *p* < 0.001) and HbO_2_ (three-way ANOVA condition × time interaction *F* = 10.2, *p* < 0.001) reduction as well as HHb increase (three-way ANOVA condition × time interaction *F* = 13.2, *p* < 0.001) were larger during SH compared to IH conditions (Table 3).

Changes in pre-frontal cortex TSI, HbO_2_, and HHb during SH and IH conditions were larger than changes at the muscle level (all *p* < 0.05).

## Discussion

The present study aimed to assess the acute and short-term (3 sessions) cardiorespiratory and tissue oxygenation responses to two hypoxic conditioning strategies, i.e., SH and IH, in order to determine their respective effects and potential physiological biomarkers able to detect early adaptations. During SH and IH sessions (acute effects), hyperventilation, tachycardia, increased blood pressure, and reduced heart rate variability were observed. SH sessions were associated with larger reduction in heart rate variability and greater muscle and prefrontal cortex deoxygenation compared to IH sessions. From S1 to S3 (short-term adaptations), IH reduced normoxic SBP and increased heart rate variability, SH sessions induced further reduction in heart rate variability, while both SH and IH sessions induced larger muscle deoxygenation. Hence, these results emphasize differences between SH and IH conditioning strategies regarding cardiovascular and tissue oxygenation responses and suggest some early adaptations after three hypoxic conditioning sessions only.

### Acute cardiorespiratory responses

Acute hypoxic exposure is known to induce an increase in ventilation within the first minutes of exposure due to stimulation of peripheral chemoreceptors Mazzeo (2008). In the present study, a significant increase in V_E_ was observed during the 5-min hypoxic phases of IH sessions but not during SH sessions (Figure 1). One potential reason for this difference between SH and IH is the progressive reduction in FiO_2_ over the first 10 min of hypoxic exposure during SH sessions while during IH subjects were switched instantaneously from normoxia to a gas mixture with a FiO_2_ of 0.10, possibly inducing greater chemoreceptor stimulation. Significant reductions in PetCO_2_ during SH and IH sessions indicate however that subjects hyperventilated during both types of hypoxic exposure (Figure 2). The hypoxic hyperventilatory response was nevertheless relatively modest since V_E_ increased by <2 L·min^−1^ and PetCO_2_ decreased by <3 mmHg on average whatever the hypoxic condition, which is consistent with previous observations during SH and IH conditioning sessions [~50-min IH with 5–6 min FiO_2_ = 0.10–4 min FiO_2_ = 0.21 (Zhang et al., 2014); 1-h SH or IH at SpO_2_ = 80–90% (Rodway et al., 2007)]. This modest increase in ventilation probably contributed to the absence of significant discomfort reported by all subjects during both SH and IH sessions.

Acute hypoxic exposure is also known to induce tachycardia as observed during both SH and IH sessions (Figure 3), due to a combination of factors including aortic bodies stimulation and modulation of the autonomous nervous system Mazzeo (2008). Changes in arterial blood pressure during acute hypoxic exposure result from the opposite effect of hypoxia-induced peripheral vasodilation (Crawford et al., 2006) and sympathetic activation (Halliwill et al., 2003). Previous studies reported inconsistent changes in arterial blood pressure during acute SH or IH exposure with no change [~50-min IH with 5–6 min FiO_2_ = 0.10–4 min FiO_2_ = 0.21 (Zhang et al., 2014)] or slight increase [1-h SH at SpO_2_ = 80% (Lusina et al., 2006), 1-h SH or IH at SpO_2_ = 80–90% (Rodway et al., 2007), 1-h IH with 5–7 min normocapnic rebreathing–5–7 min FiO_2_ = 0.21 (Bernardi et al., 2001b)]. In the present study both SH and IH induced a significant increase in blood pressure (Figure 3) which may be of larger magnitude compared to some previous studies due to the relatively low target SpO_2_ (70–80%).

Heart rate variability is generally increased during acute hypoxic exposure which is thought to reflect sympathetic activation and parasympathetic withdrawal (Roche et al., 2002). In the present study, heart rate variability decreased during SH and IH sessions (from baseline normoxic exposure) suggesting a shift in the sympathovagal balance due to sympathetic activation as suggested by the increased LF/HF ratio in SH (Figure 5) and vagal withdrawal as suggested by reduced PNN50 and RMSSD in both SH and IH (Figure 4; Malik, 1996). Because changes in ventilation were modest in the present study and more pronounced in the hypoxic condition associated with smaller changes in heart rate variability (i.e., IH), it is likely that changes in heart rate variability reflected alterations in the sympathovagal balance rather than respiratory sinus node modulation (Hirsch and Bishop, 1981). Interestingly, the reduction in heart rate variability was larger during SH compared to IH as shown by lower SDNN, PNN50, and RMSSD values during SH compared to IH sessions (Table 2). A greater effect of SH might be explained by the larger dose of hypoxia characterizing SH sessions compared to IH sessions due to the sustained hypoxic pattern of exposure (without reoxygenation phases as in IH) and the slightly lower SpO_2_ (2–3% lower at mid- and end-session) in SH compared to IH sessions (Table 1). The importance of the total hypoxic dose has also been suggested by Tamisier et al. (2005) who observed a larger increase in muscle sympathetic nerve activity after 2 h of SH (SpO_2_ ~ 85%) vs. 2 h of IH (~2-min cycles with minimum SpO_2_ ~ 85%). The more severe reduction of heart rate variability during SH compared to IH observed in the present (Table 2) and previous studies is however in contrast to previous observations suggesting greater cardiorespiratory alterations due to intermittent vs. sustained hypoxia in rats (8-h/day IH with 15 s FiO_2_ = 0.05–5 min FiO_2_ = 0.21; 4-h/day SH at ~6500 m), possibly due to oxidative stress associated with repetitive deoxygenation–reoxygenation cycles (Peng and Prabhakar, 2004). In the later study, the cycle duration and hypoxia severity were nevertheless shorter and more severe, respectively, (and therefore potentially more harmful) which may explain these contrasting results when comparing SH and IH exposures.

Under similar hypoxemic levels (i.e., similar target SpO_2_ in SH and IH), the present study demonstrates that (i) SH induces larger reductions in muscle (larger HbO_2_ and HHb but not TSI changes) and even more pre-frontal cortex (larger HbO_2_, HHb, and TSI changes) oxygenation compared to IH sessions (Table 3) and (ii) larger reductions in pre-frontal cortex than muscle oxygenation are induced during both SH and IH sessions (Figure 6). Of note, these changes in tissue oxygenation occurred together with unchanged HbTot both at the pre-frontal cortex and muscle levels (Figures 7, 8), suggesting no significant change in local blood flow. Although the slightly greater reduction in SpO_2_ during SH compared to IH (~2% difference, Table 1) may have contributed to the greater tissue deoxygenation during SH sessions, it is unlikely to explain the large difference in tissue oxygenation between both hypoxic conditions (Table 3). Since we have previously shown that both muscle and cerebral deoxygenations display slower kinetics than arterial deoxygenation when FiO_2_ is reduced (Rupp et al., 2013), it can be suggested that 5-min hypoxic phases during IH sessions did not allow maximal muscle and pre-frontal cortex deoxygenation compared to prolonged and sustained arterial oxygenation reduction characterizing SH sessions (where maximal deoxygenation required up to 30 min of hypoxic exposure, Figure 6). Whether the larger cerebral deoxygenation induced by SH compared to IH sessions contributed to some differences in cardiorespiratory responses observed between the two types of hypoxic exposure in the present study (e.g., the greater reduction in heart rate variability in SH compared to IH) remains to be elucidated.

The larger reduction in pre-frontal cortex oxygenation compared to muscle oxygenation during both SH and IH sessions (Figure 6) is in accordance with the greater cerebral oxygenation sensitivity to reduction in arterial oxygenation previously reported by our group under both sustained [FiO_2_ = 0.12 (Rupp et al., 2013)] and intermittent [100 s FiO_2_ = 0.12–20 s FiO_2_ = 1.0 (Rupp et al., 2016)] hypoxia, possibly due to greater basal metabolic activity and the inability to recruit additional capillaries at the cerebral compared to the muscle level. In order to induce significant deoxygenation and therefore potential adaptations within the muscle, coupling hypoxic exposure and physical exercise may be an attractive hypoxic conditioning strategy to consider (Millet et al., 2016).

### Short-term cardiorespiratory adaptations

While chronic hypoxic exposure such as at high altitude is known to increase the hypoxic ventilatory response [e.g., 5 days at 4350 m (Rupp et al., 2014)], repeated bouts of intermittent hypoxia have also been shown to increase hypoxic chemosensitivity [7 days, 1–3-h/day SH with FiO_2_ = 0.12 (Katayama et al., 2009); 10 days, 1-h/day SH at SpO_2_ = 80% (Lusina et al., 2006); 14 days, 1-h/day IH with 5–7 min normocapnic rebreathing—5–7 min FiO_2_ = 0.21 (Bernardi et al., 2001b); 10 days, 1-h/day IH with 5 min FiO_2_ = 0.12–5 min FiO_2_ = 0.21 (Foster et al., 2005)]. In the present study, three sessions of hypoxic conditioning did not change the ventilatory response during SH or IH (Figure 2) while previous studies reported increased hypoxic ventilatory responses after at least seven hypoxic sessions (Bernardi et al., 2001b; Foster et al., 2005; Lusina et al., 2006; Katayama et al., 2009). Changes in hypoxic ventilatory response may require a larger amount of hypoxic conditioning sessions than performed in the present study. Hypoxic heart rate response has been reported to be reduced [~50-min IH with 5–6 min FiO_2_ = 0.10–4 min FiO_2_ = 0.21, (Zhang et al., 2014)] or unchanged [7 days, 1-h/day SH at 4500 m (Katayama et al., 2001); 10 days, 1-h/day IH with 5 min FiO_2_ = 0.12–5 min FiO_2_ = 0.21 (Foster et al., 2005)] by repeated exposure to intermittent hypoxia. In the present study, the SH- or IH-induced increases in HR were similar at S1 and S3 (Figure 3), indicating no change in heart rate hypoxic response after three hypoxic conditioning sessions.

While SH induced similar increase in blood pressure during S1 and S3, a significant increase in SBP was observed in IH during S3 only, due to a reduction in baseline normoxic value compared to S1 (Figure 3). This ~8 mmHg SBP reduction is of significance in healthy young subjects and in terms of cardiovascular prevention, and may represent a true effect of IH conditioning on vascular tone. Shatilo et al. (2008) reported similar reduction in blood pressure in healthy older individuals (61 ± 2 years) following 10 days of IH conditioning (40 min/day IH with 5-min FiO_2_ = 0.12–5-min FiO_2_ = 0.21). Several studies also suggested that in patients with hypertension, hypoxic conditioning programs can significantly reduce blood pressure [see for a review (Serebrovskaya et al., 2008)]. Lyamina et al. (2011) for instance reported in patients with stage 1 hypertension a significant decrease in blood pressure (>20 and >15 mmHg systolic and DBP reduction, respectively) after 20 days of IH conditioning (30–60 min/day IH with 3 min FiO_2_ = 0.10–3 min FiO_2_ = 0.21). Hence, the present study suggests that even in healthy young subjects with normal blood pressure and no cardiovascular risk factor, early improvements in blood pressure can occur after only three IH conditioning sessions. Improvements in blood pressure after hypoxic conditioning may be due to changes in the autonomic function (with augmented parasympathetic tone), increased endothelial NO production and enhanced vasoactive factors such as vascular endothelial growth factor (Serebrovskaya et al., 2008). The results of the present study suggesting that IH conditioning may be more efficient in reducing blood pressure compared to SH conditioning are in accordance with the study by Rodway et al. (2007) showing some specific effects of IH (3 days, 1-h IH with 10 min SpO_2_ = 80–90%—10 min FiO_2_ = 0.21) compared to SH (3 days, 1-h SH with SpO_2_ = 80–90%) in young healthy subjects, i.e., an increase in nitric oxide synthases expression in circulating lymphocytes negatively correlated with blood pressure after three IH conditioning sessions only.

Interestingly, from S1 to S3, opposite changes in heart rate variability were observed in SH compared to IH conditions (Table 2). Greater reduction in overall heart rate variability (as suggested by lower SDNN) and larger vagal withdrawal (as suggested by lower PNN50 and RMSSD) were observed in SH after three sessions. Conversely, IH was associated with larger overall heart rate variability (as suggested by larger SDNN) and greater parasympathetic activity (as suggested by larger RMSSD) at S3 compared to S1. Previous studies have reported that similar IH protocols than the present study but over longer durations also increased heart rate variability [14 days, ~50-min IH with 5–6 min FiO_2_ = 0.10–4 min FiO_2_ = 0.21 (Zhang et al., 2014); 16 days, 1-h/day IH with 5 min FiO_2_ = 0.16–0.10–5 min FiO_2_ = 0.21 (Lizamore et al., 2016)], while Lusina et al. (2006) reported that 10 daily 1-h SH exposures with SpO_2_ = 80% in healthy young subjects increased muscle sympathetic nerve activity both during and after hypoxic exposure. These previous results together with the present data suggest important differences regarding changes in heart rate variability induced by repetitive SH vs. IH exposures. Improved heart rate variability and possibly increased parasympathetic activity following three sessions of IH in the present study (Figure 4) may underlie the reduction in normoxic SBP observed at S3 in this condition. Although the significance of these early and opposite changes in heart rate variability indices observed in SH and IH needs to be further investigated, the present data suggest that measurements of heart rate variability may provide useful physiological biomarkers to monitor the effects of hypoxic conditioning strategies.

From S1 to S3, and according to the study design, similar reductions in arterial oxygenation were induced in both SH and IH conditions (Figure 1). The similar reductions in pre-frontal cortex oxygenation from S1 to S3 (Figure 8) suggest that, as opposed to the effect of several days of sustained hypoxia [5 days at 4350 m (Rupp et al., 2014)] or intermittent hypoxic exposure [10 days, 1-h/day IH with 5 min FiO_2_ = 0.12–5 min FiO_2_ = 0.21 (Foster et al., 2005)], no early changes in hypoxic cerebrovascular responses were induced. In contrast to the pre-frontal cortex, muscle deoxygenation was larger in S3 compared to S1 both in SH and IH (Figure 7). The reduction in muscle TSI, an index of tissue oxygenation, reached significance at S3 only both during SH and IH (Figure 6). These results suggest that while during the first session of hypoxic conditioning the muscle is minimally exposed to hypoxia despite substantial reduction in arterial oxygenation level, the repetition of hypoxic sessions accentuates muscle deoxygenation for identical reduction in SpO_2_. This may suggest that the potential effects of hypoxic conditioning on the muscle (if any) may require several sessions to be induced. The reasons for this reduction in muscle oxygenation triggered after several hypoxic conditioning sessions remain to be determined but larger muscle sympathetic nerve activity as observed by Lusina et al. (2006) after 10 daily 1-h SH exposures with SpO_2_ = 80% may contribute to reduced muscle perfusion and oxygenation, at least in SH condition.

### Limitations

Although the present study provides several important insights into the acute and short-term responses to SH and IH conditioning strategies, several other potential adaptations remain to be evaluated such as hematological and metabolic changes or exercise cardiorespiratory responses. The effects of SH and IH conditioning protocols also need to be compared over more prolonged duration (e.g., several weeks) as usually performed to induce health benefits. The present study focused however on acute differences and early adaptations to hypoxic conditioning strategies in order to provide potential physiological biomarkers able to monitor the benefits and potential deleterious consequences of these hypoxic conditioning interventions. Females were included in the present study without taking account of potential effects of menstrual cycles on hypoxic responses. Although recent results indicate that the menstrual cycle may not affect hypoxic ventilatory responses for instance (Macnutt et al., 2012), future studies on hypoxic conditioning should clarify the effect of menstrual cycle. Finally, the present data applies to healthy young subjects and further studies are needed to evaluate the acute and short-term responses to similar hypoxic conditioning strategies in older individuals and in patients at risk of or with cardiovascular or cerebrovascular abnormalities.

### Practical considerations

The present study aimed to provide practical information useful to design hypoxic conditioning interventions for healthy subjects. These results also give a rational to further develop and investigate hypoxic conditioning interventions for patients, especially regarding the type of hypoxic exposure (SH vs. IH) and the potential physiological biomarkers that could be used during the first sessions in order to detect early adaptations. During SH sessions, larger homeostasis disruption seemed to occur compared to IH sessions, at least when considering the larger reduction in heart rate variability and greater tissue deoxygenation in SH compared to IH sessions (Tables 2, 3). Although this larger physiological stress in SH was well tolerated in the present population, it seemed to be associated with less favorable adaptations over three sessions compared to IH, at least regarding the improved heart rate variability and normoxic SBP observed from S1 to S3 in IH only. The lack of muscle deoxygenation during S1 and the relatively modest muscle deoxygenation level (compared to the pre-frontal cortex especially) at S3 suggest that both SH and IH at rest may not provide sufficient muscle homeostasis disruption in order to induce significant muscular adaptations (e.g., metabolic). Combining hypoxic exposure and muscular exercise (i.e., hypoxic exercise training) might be an attractive strategy especially to induce muscle adaptations. At last, the present study suggests that monitoring heart rate variability during and after hypoxic conditioning sessions may provide a useful physiological biomarker able to detect early adaptations and to indicate whether the hypoxic dose is adequate for a given individual. These practical considerations should help to implement hypoxic conditioning strategies to more fragile populations such as older individuals or patients with chronic diseases for instance.

## Conclusions

The present results showed significant differences in heart rate variability and tissue (especially cerebral) oxygenation changes during acute SH vs. IH exposures in healthy young subjects. In addition, after three hypoxic conditioning sessions, IH only induced significant reduction in normoxic SBP while SH and IH induced opposite effects (i.e., a reduction and an increase, respectively) on heart rate variability. Hence this study indicates that the pattern of hypoxic exposure (i.e., sustained vs. intermittent) during hypoxic conditioning intervention should be carefully selected and suggests that heart rate variability may provide useful information about the early adaptations induced by such intervention.

## Author contributions

SC, PF, SD, and SV contributed to the conception and design of the work; SC, AB, SM, SB, PF, SD, and SV contributed to data acquisition, analysis, and interpretation; SC, AB, SM, SB, PF, SD, and SV drafted the work and revised it critically; SC, AB, SM, SB, PF, SD, and SV approved the final version to be published; SC, AB, SM, SB, PF, SD, and SV agree to be accountable for all aspects of the work and ensure that questions related to the accuracy and integrity of all parts of the work are appropriately investigated and resolved.

### Conflict of interest statement

The authors declare that the research was conducted in the absence of any commercial or financial relationships that could be construed as a potential conflict of interest.
